# Assessment of potential public health impact of a quadrivalent inactivated influenza vaccine in Thailand

**DOI:** 10.1111/irv.12361

**Published:** 2016-01-29

**Authors:** Wanitchaya Kittikraisak, Malinee Chittaganpitch, Christopher J. Gregory, Yongjua Laosiritaworn, Thanawadee Thantithaveewat, Fatimah S. Dawood, Kim A. Lindblade

**Affiliations:** ^1^ Influenza ProgramThailand Ministry of Public Health – U.S. Centers for Disease Control and Prevention CollaborationNonthaburiThailand; ^2^ National Institute of HealthMinistry of Public HealthNonthaburiThailand; ^3^ International Emerging Infections ProgramThailand Ministry of Public Health – U.S. Centers for Disease Control and Prevention CollaborationNonthaburiThailand; ^4^ Division of Global Health ProtectionU.S. Centers for Disease Control and PreventionAtlantaGAUSA; ^5^ Bureau of EpidemiologyMinistry of Public HealthNonthaburiThailand; ^6^ Bureau of General Communicable DiseasesMinistry of Public HealthNonthaburiThailand; ^7^ Influenza DivisionU.S. Centers for Disease Control and PreventionAtlantaGAUSA

**Keywords:** Inactivated influenza vaccine, quadrivalent, Thailand, trivalent

## Abstract

**Background:**

Each year, an influenza B strain representing only one influenza B lineage is included in the trivalent inactivated influenza vaccine (IIV3); a mismatch between the selected lineage and circulating viruses can result in suboptimal vaccine effectiveness. We modeled the added potential public health impact of a quadrivalent inactivated influenza vaccine (IIV4) that includes strains from both influenza B lineages compared to IIV3 on influenza‐associated morbidity and mortality in Thailand.

**Methods:**

Using data on the incidence of influenza‐associated hospitalizations and deaths, vaccine effectiveness, and vaccine coverage from the 2007–2012 influenza seasons in Thailand, we estimated rates of influenza‐associated outcomes that might be averted using IIV4 instead of IIV3. We then applied these rates to national population estimates to calculate averted illnesses, hospitalizations, and deaths for each season. We assumed that the influenza B lineage included in IIV3 would provide a relative vaccine effectiveness of 75% against the other B lineage.

**Results:**

Compared to use of IIV3, use of IIV4 might have led to an additional reduction ranging from 0·4 to 14·3 influenza‐associated illnesses per 100 000 population/year, <0·1 to 0·5 hospitalizations per 100 000/year, and <0·1 to 0·4 deaths per 1000/year. Based on extrapolation to national population estimates, replacement of IIV3 with IIV4 might have averted an additional 267–9784 influenza‐associated illnesses, 9–320 hospitalizations, and 0–3 deaths.

**Conclusion:**

Compared to use of IIV3, IIV4 has the potential to further reduce the burden of influenza‐associated morbidity and mortality in Thailand.

## Introduction

The trivalent inactivated influenza vaccine (IIV3) is composed of antigens from three influenza viruses: influenza A(H1N1), influenza A(H3N2), and one of two co‐circulating lineages of influenza B virus (Victoria and Yamagata). In five of the ten Northern Hemisphere influenza seasons in the United States (US) from 1999 to 2009, the predominant B virus was not included in the seasonal vaccine.^1^, ^2^ Because vaccination with a strain from one B lineage provides variable cross‐protection against the other, a mismatch between the virus selected for the vaccine and the predominant circulating virus may reduce vaccine effectiveness.^2^ In 2012, recognizing that vaccine manufacturers were developing a quadrivalent inactivated influenza vaccine (IIV4), the World Health Organization began identifying a second influenza B strain that could be included in a seasonal influenza vaccine. However, there is currently no recommendation preferring either the trivalent or quadrivalent vaccine.^3^ Vaccine manufacturers began distributing IIV4 during the 2012–2013 Northern Hemisphere influenza season.

Many countries have modeled the potential public health or economic impact of incorporating IIV4 into the national vaccine program.^4^, ^5^, ^6^, ^7^, ^8^, ^9^, ^10^ Most studies have found a reduction in influenza‐associated morbidity and mortality from the inclusion of a second influenza B lineage.^4^, ^5^, ^6^, ^7^, ^8^, ^9^, ^10^ In the US, the potential net impact of the IIV4 on influenza‐associated outcomes was demonstrated to vary substantially between seasons, depending on the percentage of influenza virus infections caused by each of the two influenza B lineages, vaccine coverage, and vaccine effectiveness.^4^

In Thailand, annual seasonal influenza vaccination is recommended by the Ministry of Public Health for various high risk groups, including persons aged ≥65 years, persons with chronic underlying medical conditions, children aged 6 months to 2 years, pregnant women after the first trimester, healthcare personnel, mentally ill persons unable to care for themselves, and persons weighing >100 kg. Currently, IIV3 is provided free of charge in the national vaccine program (through the Ministry of Public Health and National Health Security Office) to the first five risk groups, but supply is sufficient to cover only 25% of the target population (T. Thantithaveewat personal communication) and coverage does not exceed 20% in any risk group.^11^

We conducted a model‐based analysis to estimate the potential public health impact of the IIV4 introduction on influenza‐associated morbidity and mortality in Thailand based on a model developed for the US.^4^ This model used data from Thailand from 2007 to 2012, including annual rates of influenza‐associated outcomes, the proportion of illness due to the two influenza B lineages, vaccine coverage, and the vaccine effectiveness of the Southern Hemisphere IIV3, the principal formulation used in Thailand. Given the uncertainty over the eventual price of IIV4 in Thailand, a range of price assumptions was incorporated into the model to take into account the potential for a higher IIV4 cost compared to IIV3, and a fixed national vaccine program budget.

## Methods

### Epidemiological model

**Figure 1:**
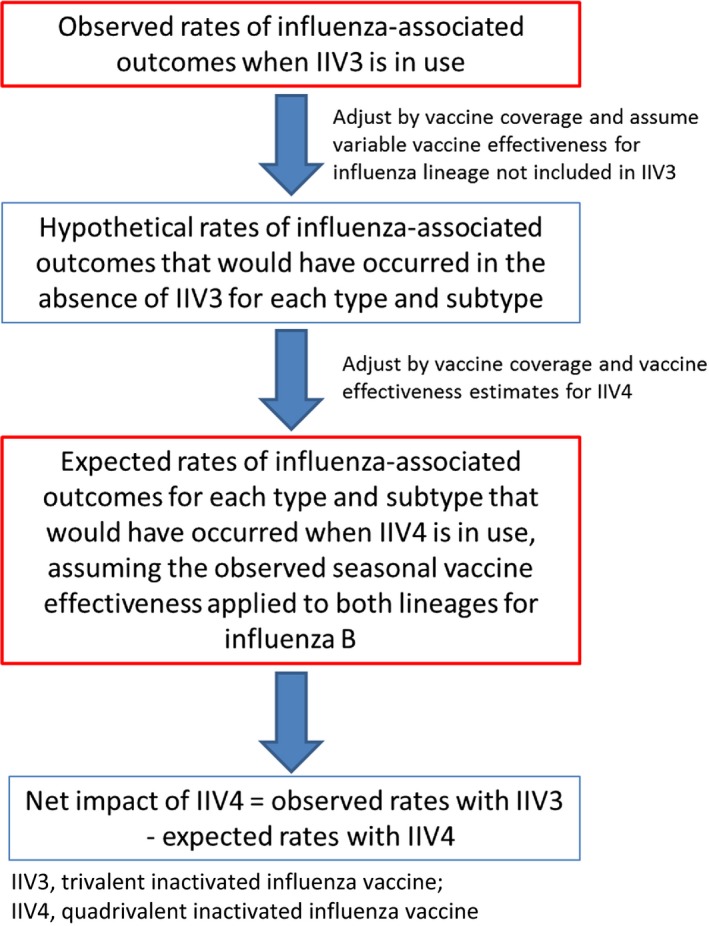
Epidemiologic concept to estimate the impact of quadrivalent inactivated influenza vaccine on influenza‐associated outcomes.

To calculate the estimated net difference in rates of influenza‐associated morbidity and mortality that would be expected from use of IIV4 in general population, observed rates of influenza‐associated illnesses, hospitalizations, and deaths during years when IIV3 was used were compared to rates modeled with use of IIV4 (Figure 1).^4^

We calculated the hypothetical rates at which influenza‐associated illnesses, hospitalizations, and deaths would have occurred in the absence of IIV3 for each type, subtype, and lineage of influenza virus using the following formula:

$$\text{Expected rate without vaccination}=\frac{\text{observed rate with IIV}3}{1-(\text{VC}\times VE_{\text{IIV3}})},$$

where VC is the vaccine coverage, and VE is the vaccine effectiveness. In base case analysis, we assumed that the influenza B lineage included in IIV3 would provide a relative vaccine effectiveness of 75% against the other lineage not included in the IIV3 formulation.^12^ We also varied this value in sensitivity analyses to accommodate different levels of cross‐protection against the other lineage that was not included. To calculate the expected rates of influenza‐associated outcomes if IIV4 had been used instead, we used the following formula:

$$\text{Expected rate with IIV4}=\text{expected rate without vaccination}\times (1-\left(\text{VC}\times {\text{VE}}_{\text{IIV4}}\right)).$$

We then calculated the annual net difference in the number of cases of influenza‐associated illness, hospitalization, and death that would be expected using IIV4 rather than IIV3 by applying the net rate differences to national population estimates. We reported estimate of each season and also ranges based on these point estimates.

### Data inputs

Table 1 shows the range of data input values and respective sources. All data were available in aggregated form and gathered from published and unpublished sources, except the observed incidence of influenza‐associated hospitalizations and deaths and influenza virologic surveillance data, for which individual‐level, anonymized data were retrieved. We consulted with 14 subject‐matter experts from a vaccine company and Thailand's Ministry of Public Health to ensure that all assumptions made and data input values (Table S1) were best suited for Thailand. To account for uncertainty of some input parameters, we considered a possible range of each input, suggested by the experts, in sensitivity analyses.

**Table 1:** Ranges of parameter estimates included in the evaluation of a quadrivalent inactivated influenza vaccine for the 2007–2012 influenza seasons in Thailand

Parameters	Range across 2007–2012 seasons	Source
Population	66–68 million	National Economic and Social Development Board, Prime Minister's Office and the Official Statistics Registration System, Department of Provincial Administration, Ministry of Interior^13^, ^14^
Incidence of influenza‐associated hospitalizations and deaths	Hospitalization: 59–118 per 100 000 population Death: 0·5–1·6 per 100 000 population	Population‐based surveillance for hospitalized lower respiratory tract infections, International Emerging Infections Program, Thailand MOPH – U.S. CDC Collaboration (the surveillance was described in Olsen *et al*. and Baggett *et al*.)^18^, ^19^
Incidence of influenza‐associated illnesses	Illness: 1541–5487 per 100 000 population	Calculated from incidence of influenza‐associated hospitalization and case fatality ratio^8^, ^20^
Distribution of influenza type, subtype, and lineage	B (any): 12–53% B/Yamagata: 3–61% B/Victoria: 39–97%	National virologic surveillance, National Institute of Health, Ministry of Public Health (data are available on http://www.thainihnic.org/influenza/main.php and additional unpublished data were retrieved)
Vaccine effectiveness (against laboratory‐confirmed medically attended influenza virus infection) for trivalent inactivated influenza vaccine	25–58%	Sullivan *et al*.^21^ (2012 influenza season) Kittikraisak *et al*.^22^ (2011–2012 influenza seasons) Levy *et al*.^23^ (2010–2012 influenza seasons) Kelly *et al*.^24^ (2007–2011 influenza seasons) Dawood *et al*.^25^ (2010 influenza season) Madhi *et al*.^26^ (2008 influenza season) Orellano *et al*.^27^ (2009 influenza season)
Doses of trivalent inactivated influenza vaccine imported	2·0–8·9 million	Food and Drug Administration, Ministry of Public Health (data were published in Gupta *et al*. and additional unpublished data were retrieved)^17^
Doses of trivalent inactivated influenza vaccine administered	1·9–8·4 million	Calculated from annual doses of trivalent inactivated influenza vaccine imported and estimated wastage rate^11^
Vaccination coverage in general population	3–12%	Calculated from vaccine doses administered and total population ≥6 months of age

### Population data

We obtained population data from the National Economic and Social Development Board of Thailand.^13^ Because this population source reports population figures by 5‐year age increments, we used data from the Ministry of Interior, which reports the population in finer age intervals (<1 and ≥1 years), to calculate the proportion of children aged <6 months (by dividing the proportion of children aged <1 year by two).^14^ We applied this proportion to the total population to get an estimated number of children aged <6 months. To calculate the population eligible for influenza vaccination, we subtracted the number of children aged <6 months from the total population.

### Influenza season and influenza vaccine

In Thailand, influenza viruses circulate year‐round with a peak during June–November (when approximately 60% of the cases occur)^15^ and a smaller peak during January–March.^16^ Because seasonal influenza vaccination in Thailand is recommended to start in May, we defined an influenza season as June through May of the following year (e.g. the 2012 influenza season is June 2012‐May 2013).^15^ Evidence indicates that protection against viruses that are antigenically similar to those contained in the vaccine declines after 6–8 months.^3^ All seasonal influenza vaccines licensed and used in Thailand from 2007 to 2012 seasons were IIV3. Although both Northern and Southern Hemisphere vaccines were available in Thailand, all vaccines purchased by the government and provided free of charge (approximately half the supply), as well as the majority of the privately purchased vaccines, were the Southern Hemisphere formulation.^17^ Therefore, estimates of vaccine effectiveness used in the model were from the Southern Hemisphere IIV3.

### Incidence of influenza‐associated outcomes

The observed incidence of influenza‐associated hospitalizations and deaths was measured from active population‐based surveillance for hospitalized acute lower respiratory tract infections in two provinces in Thailand.^18^, ^19^ We chose to use the data from this source instead of the national passive surveillance system (based on discharge diagnoses classified according to the International Classification of Diseases 10) as data from the latter source are rarely laboratory‐confirmed and likely to underestimate true disease rates. An influenza‐associated hospitalization was defined as a respiratory illness requiring hospitalization with a specimen positive for an influenza virus by polymerase chain reaction. The influenza‐associated mortality rate was estimated from in‐hospital deaths among patients with influenza‐associated hospitalizations.

The incidence of influenza‐associated illnesses was not measured directly but was back‐calculated from the influenza‐associated mortality rate by applying the case fatality ratio (0·03%) reported through the national passive surveillance system, averaged across 14 years.^8^, ^20^

### Distribution of influenza type, subtype, and lineage

The annual rates of influenza‐associated outcomes by type, subtype, and lineage of influenza were calculated based on the distribution of influenza viruses identified through the national influenza virologic surveillance, recalculated by influenza season.^16^ These proportions were applied to the incidence of influenza‐associated illnesses, hospitalizations, and deaths to calculate rates by type and subtype or lineage.

### Vaccine effectiveness

The vaccine effectiveness of the Southern Hemisphere IIV3 was estimated from published data from countries that used Southern Hemisphere vaccines.^21^, ^22^, ^23^, ^24^, ^25^, ^26^, ^27^ The estimated vaccine effectiveness by influenza season was calculated by averaging all vaccine effectiveness data from a given season. Because there are no published data on the effectiveness of the Southern Hemisphere IIV4, we used data on the effectiveness of Southern Hemisphere IIV3 and assumed this level of effectiveness would apply to both lineages of influenza B included the IIV4.^4^, ^5^

### Doses of vaccine available, administered, and vaccination coverage

Data on the number of doses of influenza vaccine imported into the country were obtained from the Food and Drug Administration, Ministry of Public Health.^17^ Influenza vaccine wastage rates in the public setting from 2010 to 2012 were from the published literature.^11^ Because our study covered a wider range of influenza seasons, we used an average wastage rate of 9·5% for the seasons with unavailable wastage data. To calculate the number of doses administered, we multiplied the number of IIV3 doses sold by 1‐wastage rate. The wastage rates of vaccine used in private setting were not available; in consultation with subject‐matter experts, we used a lower wastage rate of 3·0% based on the assumption that the private vaccine providers who paid for the vaccines were unlikely to waste them. We applied this 3·0% wastage rate in the private setting to the number of IIV3 doses sold privately to estimate the number of doses administered in this setting. We then divided the number of the IIV3 doses administered (both in public and in private settings) by the population aged ≥6 months to estimate the vaccine coverage in the general population.^13^

### Sensitivity analyses

We altered some model inputs in sensitivity analyses in which we (i) used a case fatality ratio of 0·05% to calculate the incidence of influenza‐associated illnesses,^20^ (ii) used incidence of influenza‐associated illness of 5% and 10%,^28^, ^29^ (iii) used estimates of vaccine effectiveness to prevent influenza‐associated outcomes from the influenza B lineage not included in the IIV3 formulation of 0%, 10%, 20%, and 30%, and (iv) used vaccine wastage rates of 15% and 20% in public sector and of 10% and 15% in private sector, while keeping the values of other parameters fixed.

### Potential price increase and budget

As the cost of IIV4 in Thailand was not available at the time of analysis, we calculated the reduction in vaccine coverage that would result from a 0% to 30% increase in the price of IIV4 over IIV3, assuming a fixed national budget for influenza vaccine purchase. Based on a comparison of the net difference in influenza‐associated illnesses, hospitalizations, and deaths among various price assumptions, we estimated the price increase threshold below which IIV4 would continue to provide public health impact. We reported the threshold for which use of IIV4 would continue to avert at least one additional influenza‐associated outcome compared to use of IIV3. The currency exchange rate used in the analysis was 35 Thai Baht to 1 USD.

## Results

### Distribution of influenza strains

The proportion of influenza B among all influenza viruses identified in Thailand was highest in 2007 (52·7%) and lowest in the 2009 season (12·0%; Table 2). During 2007–2012, both influenza B lineages (i.e. Victoria and Yamagata) circulated concurrently but with varying predominance. The Victoria lineage was predominant from 2007 to 2011, while the Yamagata lineage was predominant in 2012. In three of the six influenza seasons (2008, 2009, and 2012 seasons), the predominant circulating influenza B lineage was different from the lineage included in the Southern Hemisphere IIV3.

**Table 2:** Distribution of influenza B viruses by lineage and season from the national virologic surveillance, Thailand, for the 2007–2012 influenza seasons

Season	% of influenza B among all influenza viruses identified	% of influenza B from Yamagata lineage	% of influenza B from Victoria lineage	Lineage included in Southern Hemisphere trivalent inactivated influenza vaccine	Match of predominant epidemic strain to vaccine strain?
2007	52·7	20·8	79·2	Victoria	Yes
2008	28·7	27·8	72·2	Yamagata	No
2009	12·0	2·8	97·2	Yamagata	No
2010	40·3	2·7	97·3	Victoria	Yes
2011	40·9	10·2	89·8	Victoria	Yes
2012	47·2	60·7	39·3	Victoria	No

### Net difference in rates of influenza‐associated outcomes

The observed annual incidence of influenza‐associated illnesses, hospitalizations, and deaths when IIV3 was used from 2007 to 2012 ranged from 1541·06 to 5487·39 per 100 000 population, 59·41 to 118·42 per 100 000 population, and 46·23 to 164·62 per 1000 population, respectively (Table 3). The modeled annual incidence of influenza‐associated illnesses, hospitalizations, and deaths if IIV4 had been used instead ranged from 1540·11 to 5483·10 per 100 000 population, 59·37 to 118·32 per 100 000 population, and 46·20 to 164·49 per 1000 population, respectively.

**Table 3:** Difference in influenza‐associated outcomes between the expected incidence with a quadrivalent inactivated influenza vaccine (IIV4) and the observed incidence with trivalent inactivated influenza vaccine (IIV3) in Thailand, by influenza season^a^

Season	Illness				Hospitalization				Death			
	Incidence per 100 000 population			Difference in number^d^	Incidence per 100 000 population			Difference in number	Incidence per 1000 population			Difference in number
	IIV3^b^	IIV4^c^	Difference		IIV3	IIV4	Difference		IIV3	IIV4	Difference	
2007	1541·06	1540·11	−0·95	−634	59·41	59·37	−0·04	−24	46·23	46·20	−0·03	−0·2
2008	2806·61	2800·69	−5·93	−3961	107·15	106·92	−0·23	−151	84·20	84·02	−0·18	−1·2
2009	3740·12	3733·55	−6·57	−4418	110·66	110·47	−0·19	−131	112·20	112·01	−0·20	−1·3
2010	3303·32	3302·93	−0·40	−267	109·03	109·02	−0·01	−9	99·10	99·09	−0·01	−0·1
2011	5487·39	5483·10	−4·29	−2910	118·42	118·32	−0·09	−63	164·62	164·49	−0·13	−0·9
2012	2842·16	2827·81	−14·35	−9784	93·07	92·60	−0·47	−320	85·26	84·83	−0·43	−2·9
Total for 2007–2012				−21 974					−698			−6·6

a Assuming no loss in purchasing power (dose for dose replacement of IIV3 with IIV4) and influenza B lineage included in IIV3 would provide a relative vaccine effectiveness of 75% against the other lineage not included in the IIV3 formulation.

b Observed incidence when IIV3 was in use.

c Modeled incidence if IIV4 had been used instead.

d Negative numbers indicate net influenza‐associated outcomes averted with IIV4 compared to IIV3.

In Thailand, assuming that the influenza B lineage included in IIV3 would provide a relative vaccine effectiveness of 75% against the other B lineage, a dose for dose replacement of IIV3 with IIV4 might result in further reduction in cases of influenza‐associated illness (annual additional cases averted ranged from 267 to 9784), hospitalization (annual additional hospitalizations averted ranged from 9 to 320), and death (annual additional deaths averted ranged from 0 to 3) compared to when IIV3 was used (Table 3). The net impact of IIV4 was highest in years when the predominant circulating influenza B strain was not represented in the IIV3. The additional reduction in annual rates of influenza‐associated outcomes ranged from 0·40 to 14·35 illnesses per 100 000 population, 0·01 to 0·47 hospitalizations per 100 000 population, and 0·01 to 0·43 deaths per 1000 population. Overall, we estimated that IIV4 could have averted an additional 21 974 cases of illness (average, 5·41 per 100 000 population; 95% confidence interval [95% CI], 1·37 to 9·46 per 100 000 population), 698 influenza‐associated hospitalizations (average, 0·17 per 100 000 population; 95% CI, 0·04 to 0·31 per 100 000 population), and seven influenza‐associated deaths (average, <0·01 per 1000 population; 95% CI, 0 to <0·01 per 1000 population) compared to when IIV3 was used.

Compared to IIV3, there was a consistent positive net benefit from use of IIV4 across a range of assumptions for model parameters. The magnitude of the impact was increased when the parameters were varied in all sensitivity analyses (Table S2).

### IIV4 price increase and budget scenarios

The cost of IIV3 is 3·71 USD per dose in the public setting with bulk purchase and 11·43 USD in the private setting. Assuming a fixed budget and that the influenza B lineage included in IIV3 provided 75% effective cross‐protection against the other B lineage that was not included, the maximum IIV4 price increase threshold for which the vaccine would continue to provide additional public health impact over IIV3 was 9% (4·05 USD per dose through bulk purchase in the public setting and 12·54 USD in the private setting) (Table 4). When 0–30% absolute cross‐protection was assumed, the maximum IIV4 price increase threshold was estimated to be 13–22%.

**Table 4:** Maximal quadrivalent inactivated influenza vaccine (IIV4) price increase thresholds that would still maintain the benefit of IIV4 over trivalent inactivated influenza vaccine (IIV3), given differing cross‐protection levels for lineage not included in IIV3^a^

Level of cross‐protection	% increase in IIV4 price compared to IIV3 price	Expected IIV4 price calculated based on % increment in price of IIV3		Cumulative influenza‐associated outcomes averted with IIV4 compared to IIV3 (2007–2012)		
		Price of IIV3 of 3·7 USD per dose^b^	Price of IIV3 of 11·4 USD per dose^c^	Illness	Hospitalization	Death
75% of lineage included in IIV3 formulation	8	4·01	12·31	−10 222	−357	−3
	**9**	4·05	12·43	−4603	−193	−1
	10	4·09	12·54	914	−33	0
	11	4·12	12·65	6331	125	2
None	20	4·46	13·68	−11 462	−564	−3
	21	4·49	13·79	−6936	−432	−2
	**22**	4·53	13·91	−2483	−302	−1
	23	4·57	14·02	1897	−175	1
	24	4·61	14·14	6207	−50	2
	25	4·64	14·25	10 448	73	3
10% absolute cross‐protection	17	4·35	13·34	−11 821	−536	−4
	18	4·38	13·45	−7053	−398	−2
	**19**	4·42	13·57	−2366	−261	−1
	20	4·46	13·68	2244	−127	1
	21	4·49	13·79	6777	5	2
20% absolute cross‐protection	14	4·23	13·00	−12 669	−523	−4
	15	4·27	13·11	−7642	−376	−2
	**16**	4·31	13·22	−2700	−233	−1
	17	4·35	13·34	2156	−92	1
	18	4·38	13·45	6931	47	2
30% absolute cross‐protection	12	4·16	12·77	−8754	−370	−3
	**13**	4·20	12·88	−3538	−218	−1
	14	4·23	13·00	1,586	−69	0
	15	4·27	13·11	6622	77	2

a Assuming fixed budget; negative numbers indicate net influenza‐associated outcomes averted with IIV4 compared to IIV3 (2007–2012); bold type indicates the thresholds for which use of IIV4 could have averted at least one additional influenza‐associated outcome compared to when IIV3 was in use.

b 35 Baht = 1 USD, approximated price of IIV3 with bulk purchase.

c 35 Baht = 1 USD, approximated price of IIV3 in private setting.

## Conclusions

Using a simple model, we explored the potential public health impact of IIV4 versus IIV3 for preventing influenza‐associated illnesses, hospitalizations, and deaths in Thailand. Our findings indicate that the influenza disease burden for all seasons from 2007 to 2012 would have been decreased by use of IIV4 if vaccine coverage had been maintained at the same level as IIV3; the impact was greatest for influenza‐associated illnesses and hospitalizations but minimal against influenza‐associated deaths likely due to an underestimation of in‐hospital mortality data. When considering various price assumptions and a fixed national vaccine program budget, we estimated that the maximum price increase that could be supported for IIV4 that still would have averted at least one additional influenza‐associated outcome was 9–22% depending on assumptions regarding the level of influenza B cross‐protection.

The potential public health impact of the IIV4 was estimated previously in the United Kingdom, US, Germany, China, and Canada.^4^, ^5^, ^6^, ^7^, ^8^, ^9^, ^10^ Our findings are consistent with other reports that estimated a reduction in influenza B‐associated outcomes by the IIV4 in years when the predominant circulating influenza B lineage was not included in the vaccine formulation. In our analysis, the estimated potential impact of IIV4 was substantial in the years when a significant mismatch between the vaccine and circulating strains occurred. We also found that a dose for dose replacement of IIV3 with IIV4 appeared to be beneficial for all influenza seasons from 2007 to 2012 in contrast to the US where the benefit of IIV4 varied by season.^4^ One explanation for this finding could be the perennial transmission of influenza in Thailand compared to the seasonal transmission in the US.

Estimates of the incidence of influenza‐associated outcomes (illness, hospitalization and death) were subject to some limitations. Incidence rates for hospitalizations and deaths were measured in just two provinces in Thailand and generalized to the entire country, but may not accurately reflect disease burden in other provinces. Nonetheless, we believe these estimates, derived from active population‐based surveillance, are more accurate than the national data collected from passive surveillance system which, by nature, are limited by variability and incompleteness in reporting.^30^ The incidence rates for influenza‐associated illnesses were not directly measured but estimated from observed hospitalization rates and a case fatality ratio measured through passive surveillance. While the estimated rates for Thailand (range, 1541–5487 per 100 000 population) appeared to be comparable with those reported from the US (range, 3000–10 000 per 100 000 population),^4^ biases in the measurement of a case fatality ratio for influenza through passive surveillance could have affected the accuracy of our estimation of incidence rates. We found that the calculated incidence of influenza‐associated illnesses in our study were slightly lower (range, 1–5%) than what was reported in a recent meta‐analysis and the World Health Organization's (WHO's) estimation.^31^, ^32^ Our sensitivity analyses showed a greater impact on the burden of influenza‐associated illnesses for a dose for dose replacement of IIV3 with IIV4 when the WHO's incidence of 5% and 10% were used.^32^ Further, we believe that a mortality rate based on hospital‐based surveillance is likely to underestimate mortality because it will not include out‐of‐hospital mortality and mortality that might occur from influenza‐associated complications after virus is no longer detectable. The mortality rates derived from the surveillance were indeed lower than recent reports from Thailand by Cooper *et al*. 33 and Aungkulanon *et al*.^34^ (6·1 annual deaths per 100 000 population for 2005–2009 and 4·9 deaths per 100 000 population for 2006–2011). Based on the model presented here, higher mortality estimates for influenza would result in a greater reduction in the burden of influenza‐associated mortality for a dose for dose replacement of IIV3 with IIV4.

Reviewing the published literature to gather input parameters for the model highlighted important gaps in influenza knowledge in Thailand. In particular, we found few estimates of vaccine effectiveness for countries in the Southern Hemisphere in general, and Thailand in particular. There were only two published articles and one abstract that reported the effectiveness of Southern Hemisphere IIV3 used in Thailand, and these reports were limited to key risk groups.^22^, ^25^, ^35^ Population‐based estimates of influenza incidence in Asia are also limited.^36^

Our model has a few limitations. First, we could only investigate the public health impact of IIV4 for six influenza seasons due to the lack of high‐quality data for other seasons. Second, the model did not take into account the impact of herd immunity, although the estimated vaccination coverage for all target groups is low in Thailand, making it likely that herd immunity effects are minimal. Further, the vaccine effectiveness estimates used in the model were against laboratory‐confirmed medically attended influenza virus infection and we did not apply vaccine effectiveness against mortality when we considered the benefit of IIV4 for such outcome. It is likely that the effectiveness of the influenza vaccine in preventing mortality might differ and, therefore, we may inaccurately estimate the potential impact of IIV4 on mortality. Nonetheless, the base case analysis using 75% relative vaccine effectiveness assumption addressed recent findings that suggested vaccine effectiveness for the opposite B lineage may be better than previously expected.^12^, ^37^

The adapted model in this study was aimed to be simple yet informative to permit updates to include other scenarios (e.g. risk group specific, budget circumstances) should more data become available or future influenza seasons. Our findings support a net positive public health impact from IIV4 implementation in Thailand. Assuming limited flexibility in the available budget for influenza vaccine purchase, policymakers could make use of the pricing threshold data to inform decisions about an acceptable IIV4 price range.

## Financial disclosure

All authors declare they have no financial relationships relevant to this article to disclose.

## Disclaimer

The findings and conclusions in this report are those of the authors and do not necessarily represent the official position of the U.S. Centers for Disease Control and Prevention.

## Supporting information


**Table S1.** Data input values used in the assessment of potential public health impact of a quadrivalent inactivated influenza vaccine in Thailand.
**Table S2.** Difference in influenza‐associated outcomes between that expected with a quadrivalent inactivated influenza vaccine (IIV4) and that observed with trivalent inactivated influenza vaccine (IIV3) in Thailand in various scenarios.
