# Supplementary material for: Assessment of potential public health impact of a quadrivalent inactivated influenza vaccine in Thailand
Source: Influenza Other Respir Viruses. 2016 Jan 29;10(3):211–9. doi: 10.1111/irv.12361 (PMC4814859; doi:10.1111/irv.12361)
Supplement: Supplementary file 1 — Table S1. Data input values used in the assessment of potential public health impact of a quadrivalent inactivated influenza vaccine in Thailand. Table S2. Difference in influenza‐associated outcomes between that expected with a quadrivalent inactivated influenza vaccine (IIV4) and that observed with trivalent inactivated influenza vaccine (IIV3) in Thailand in various scenarios. [file IRV-10-211-s001.docx]

**Supplemental table 1:** Data input values used in the assessment of potential public health impact of a quadrivalent inactivated influenza vaccine in Thailand

| **Season** | **2007** | **2008** | **2009** | **2010** | **2011** | **2012** |
| --- | --- | --- | --- | --- | --- | --- |
| Population | 66,420,662 | 66,841,427 | 67,250,836 | 67,534,711 | 67,849,212 | 68,188,742 |
| Incidence of influenza-associated hospitalization per 100,000 population | 59 | 107 | 111 | 109 | 118 | 93 |
| Incidence of influenza-associated death per 100,000 population | 0.5 | 0.8 | 1.1 | 1 | 1.6 | 0.9 |
| Incidence of influenza-associated illnesses per 100,000 population | 1,541 | 2,807 | 3,740 | 3,303 | 5,487 | 2,842 |
| Distribution of influenza A | 47.3% | 71.3% | 88.0% | 59.7% | 59.1% | 52.8% |
| Distribution of influenza B | 52.7% | 28.7% | 12.0% | 40.3% | 40.9% | 47.2% |
| % H1N1 | 43.4% | 57.2% | 88.2% | 74.1% | 6.8% | 53.6% |
| % H3N2 | 56.6% | 42.8% | 11.8% | 25.9% | 93.2% | 46.4% |
| % Yamagata | 20.8% | 27.8% | 2.8% | 2.7% | 10.2% | 60.7% |
| % Victoria | 79.2% | 72.2% | 97.2% | 97.3% | 89.8% | 39.3% |
| Vaccine effectiveness (95% confidence interval) | 58% (17-79%) | 52% (5-84%) | 25% (20-39%) | 57% (32-84%) | 55% (1-81%) | 55% (11-76%) |
| Doses of trivalent inactivated influenza vaccine imported | 2.0 | 4.2 | 6.8 | 7.3 | 8.0 | 9.0 |
| Doses of trivalent inactivated influenza vaccine administered | 1.9 | 4.0 | 6.5 | 6.9 | 7.5 | 8.5 |
| Vaccination coverage in general population | 2.8% | 6.0% | 9.6% | 10.1% | 11.1% | 12.4% |

**Supplemental table 2:** Difference in influenza-associated outcomes between that expected with a quadrivalent inactivated influenza vaccine (IIV4) and that observed with trivalent inactivated influenza vaccine (IIV3) in Thailand in various scenarios.^a^

| Scenario | Cumulative influenza-associated outcomes averted with IIV4 compared to IIV3 (2007-2012)^b^ | | |
| --- | --- | --- | --- |
|  | Illness | Hospitalization | Death |
| Base case analysis | -21,974 | -698 | -7 |
| Case fatality ratio of 0.05% was used to calculate incidence of influenza-associated illnesses | -50,401 | -2,673 | -25 |
| Incidence of influenza-associated illness of 5% was used | -135,084 | -11,281 | -133 |
| Incidence of influenza-associated illness of 10% was used | -270,168 | -23,302 | -175 |
| Vaccine effectiveness of influenza B lineage not included in the IIV3 formulation of 0% was assumed | -84,001 | -2,673 | -25 |
| Vaccine effectiveness of influenza B lineage not included in the IIV3 formulation of 10% was assumed | -70,397 | -2,239 | -21 |
| Vaccine effectiveness of influenza B lineage not included in the IIV3 formulation of 20% was assumed | -56,503 | -1,797 | -16 |
| Vaccine effectiveness of influenza B lineage not included in the IIV3 formulation of 30% was assumed | -42,311 | -1,346 | -13 |
| Vaccine wastage rate in public sector of 15% was assumed | -82,034 | -2,610 | -25 |
| Vaccine wastage rate in public sector of 20% was assumed | -80,246 | -2,553 | -24 |
| Vaccine wastage rate in private sector of 10% was assumed | -80,275 | -2,554 | -24 |
| Vaccine wastage rate in private sector of 15% was assumed | -77,614 | -2,470 | -23 |

^a^Only the mentioned parameter was changed for each scenario, other parameters were kept fixed

^b^Negative numbers indicate net influenza-associated outcomes averted with IIV4 compared to IIV3
